# Volatile Acid-Solvent Evaporation (VASE): Molecularly Homogeneous Distribution of Acyclovir in a Bioerodable Polymer Matrix for Long-Term Treatment of Herpes Simplex Virus-1 Infections

**DOI:** 10.1155/2018/6161230

**Published:** 2018-09-26

**Authors:** James R. Stegman, Jill K. Badin, Kaitlyn A. Biles, Thamar Etienne, Sogand Fartash-Naini, Ariel D. Gordon, Zachary W. Greeley, Benjamin W. Harding, Ricardo J. Mack, Danielle Masica, Ashley N. Nelson, Amandeep K. Samra, Sarah E. Smith, Gabrielle P. Thomas, Haley J. Zack, Timothy J. Brunker, Barry J. Margulies

**Affiliations:** ^1^Towson University Herpes Virus Lab, Department of Biological Sciences, Towson University, Towson, MD 21252, USA; ^2^Department of Chemistry, Towson University, Towson, MD 21252, USA; ^3^Molecular Biology, Biochemistry, and Bioinformatics Program, Towson University, Towson, MD 21252, USA; ^4^Department of Pharmacology and Molecular Sciences, The Johns Hopkins University School of Medicine, Baltimore, MD 21205, USA

## Abstract

Treatment for herpes simplex virus-1 and -2 (HSV-1 and -2) patients who suffer from recurrent outbreaks consists of multiple daily doses of the antiviral drugs acyclovir (ACV), penciclovir, or their more orally bioavailable derivatives valacyclovir or famciclovir. Drug troughs caused by missed doses may result in viral replication, which can generate drug-resistant mutants along with clinical sequelae. We developed a molecularly homogeneous mixture of ACV with the bioerodable polymer polycaprolactone. Through scanning electron microscopy, infrared spectroscopy, gel permeation chromatography, 1H NMR, and differential scanning calorimetry, our method of combining drug and polymer, termed Volatile Acid-Solvent Evaporation (VASE), does not compromise the integrity of polymer or drug. Furthermore, VASE creates materials that deliver therapeutic amounts of drug consistently for approximately two months. Devices with high enough drug loads diminish primary infection of HSV-1 in Vero cells to the same level as seen with a single dose of ACV. Our data will lead to further experiments in animal models, demonstrating efficacy in preventing reactivation of these viruses with a single intervention, and with other antiviral drugs amenable to such manipulation. Additionally, this type of treatment would leave no trace after its useful lifetime, as drug is released and polymer matrix is degraded* in vivo*.

## 1. Introduction

The human herpes simplex virus type-1 (HSV-1) is an alphaherpesvirus in the genus* Simplexvirus* [1]. HSV-1 typically infects mucosal and dermal epithelial cells, generally causing lesions of either the lips or nose, known as cold sores or fever blisters, or genital lesions [1, 2]. It is estimated that 80% of the adult population carries HSV-1, typically asymptomatically, with primary oral infection usually occurring during childhood [1, 3]. During the latent state, viral DNA is present in the trigeminal ganglia, but no signs or symptoms of infection are present [4, 5].

Many individuals never see emergence of disease from the latent stage of infection; however, others have recurrent outbreaks. Reactivation of oral herpes occurs in an average of 33% of those infected with HSV-1 [2]. Of those who do see a recurrence, 5% have recrudescence rates of at least one episode per month, 34% have at least one episode every two to eleven months, and 61% have at least one episode per year [1, 6]. Reactivation of the virus may be attributed to many factors, including burns, physiological and emotional stress, fever, hormonal changes, and exposure to ultraviolet light [1]. In immunocompromised individuals outbreaks can occur with increased frequency and are more difficult to control [7–9].

Several different treatments are available for combating human herpes virus infections. Therapies focus on either treatment of acute symptoms or long-term suppression of the virus from reactivation. Most antiherpetic treatments are composed of multiple doses of a nucleoside analogue, such as acyclovir (ACV), penciclovir, or their more orally bioavailable derivatives valacyclovir and famciclovir, respectively [10, 11]. These drugs are effective, but require a high level of patient compliance due to relatively poor bioavailability [12–15] and relatively short* in vivo* half-life [10, 11, 16]. This requires patients to take several oral doses daily at set times to obtain constant drug levels [17].

A subcutaneous implant releasing a constant, controlled, continuous dosage of drug for an extended period of time would negate these difficulties. Compared to treating acute symptoms, suppressing reactivation of the virus by continuous, long-term daily dosing of ACV is possible [17, 18]. Such a regimen is advantageous because it reduces the pain and stress the patient may incur, keeps the virus from replicating, prevents the emergence of ACV-resistance mutants [19, 20], and reduces the chances of transmission [17].

Previously, our lab has obtained a near zero-order release of acyclovir using silicone as a matrix for long-term subcutaneous delivery of ACV [21]. This methodology also prevented recurrences of HSV-1 in an animal model [21]. Here we report a novel methodology called VASE (Volatile Acid-Solvent Evaporation) that results in a molecularly homogeneous mixture of drug and biodegradable polymer that generates long-term, consistent delivery of suppressive levels of ACV. Polycaprolactone (PCL) was chosen as the matrix material because: (i) it is one of a small number of biodegradable polymers previously approved by the FDA for other human health applications such as suture coatings and bioadhesives [22, 23], (ii) it has an expected* in vivo* half-life of nearly six months, making it useful for a comparable or longer time than other available polymers [22, 23], and (iii) its melting temperature is well within the range of keeping antiherpetics stable (the melting point of ACV is 256°C, but ACV loses antiviral activity when pretreated at temperatures higher than 80°C for 20 minutes; data not shown) [22, 23]. VASE is also predicted to increase the stability of the polymer-drug mixture when compared to previous fabrication methods [24, 25].

## 2. Materials and Methods

### 2.1. Device Development

Devices were composed of a matrix of PCL (#440752, Mn 10000; Sigma-Aldrich, St. Louis, MO) and powdered ACV (Advanced Scientific, Ft. Lauderdale, FL), combined as below.

For methodology described as “Suspension of Insoluble Drug” (SID), PCL was dissolved completely in acetonitrile (Thermo-Fisher, Waltham, MA) (35% w:v) with stirring at 65°C. ACV was added and the solution was stirred overnight or until all the solvent had evaporated in a chemical fume hood. The dried material was then subjected to 48 h in a CentriVap Complete (Labconco, Kansas City, MO) to remove any residual solvent. The resulting material was frozen in liquid nitrogen and ground with a mortar and pestle to a fine powder. This powder was then melted at 75°C in a CombiTip 25 (Eppendorf, Mt. Laurel, NJ), extruded into a 10–gauge hollow stainless steel needle (Painful Pleasures, Hanover, MD), and allowed to cool and solidify overnight at room temperature. Rods of 2-mm diameter were pushed out of the needles and cut into either 7-mm or 15-mm lengths with a razor blade.

For methodology described as “Volatile Acid-Solvent Evaporation” (VASE), similar steps were taken as those in SID except formic acid (88-97%, Acros Organics/Thermo-Fisher) (20% v:v of acetonitrile) was added to the solvent in a dropwise fashion with stirring after addition of the drug until both the drug and PCL were completely dissolved. Both the formic acid and acetonitrile were allowed to evaporate overnight and thoroughly dried as above. The resulting powder was dried and processed as above (grinding, melting, and extrusion) to create similarly shaped 15-mm or 7-mm × 2-mm diameter rods; the 15-mm rods matched the dimensions of previously engineered silicone-based rods [21, 26, 27]; note that the surface area of two 7-mm rods equaled that of a single 15-mm rod.

Several different ratios of drug:polymer (0:100, 10:90, 30:70, or 50:50 w:w) were used for characterization of SID and VASE fabrication methods. Regardless the drug:polymer ratio, all 2 mm × 15 mm devices (or pair of 2 mm × 7 mm devices) weighed 0.050 g; therefore, for example, 30:70 w:w devices typically contained 15 mg ACV and 35 mg PCL.

### 2.2. Electron Microscopy and Fourier-Transform Infrared (FTIR) Spectroscopy

SID- and VASE-created devices were cross sectioned by slicing the rods with a razor blade. These cross sections were attached to aluminum stubs via carbon sticky tabs and coated with 20 nm AuPd. Stubs were viewed and digital images captured at 1 kV on a Leo 1530 FESEM (Cambridge, UK).

PCL, SID-created 0:100 w:w ACV:PCL devices, and SID-created 30:70 w:w ACV:PCL devices were ground to fine powders in liquid nitrogen. These powders and ACV powder were subjected to FTIR on a ThermoFisher Nicolet iS10 FT-IR spectrometer fitted with a Smart iTR Attenuated Total Reflectance sampling accessory. Spectra were compared to standards to identify functional groups [28, 29].

### 2.3. Gel Permeation Chromatography

Samples of untreated PCL, SID-treated PCL, or VASE-treated PCL powders were solvated to approximately 10 mg/mL using 94% tetrahydrofuran (THF), 5% dimethyl sulfoxide (DMSO), 1% piperidine, and a trace amount of butylated hydroxytoluene (BHT). A 100 *μ*L bolus was injected for analysis into a GPC instrument (Waters Corp., Millford, MA) using an autosampler at 1 mL/min and separated using 3 THF Styragel columns in series (37.8 × 300 mm). The molecular weight was determined via interpolation using polystyrene standards.

### 2.4. Nuclear Magnetic Resonance

Untreated PCL, ACV, and VASE-created 30:70 (w:w) ACV:PCL powders were dissolved in acetonitrile-d3 (CD_3_CN), dimethyl sulfoxide-d6 (DMSO), or a 50:50 mix of those two solvents (Acros Organics/Thermo-Fisher). Tetramethylsilane (TMS) (Acros Organics/Thermo-Fisher) was added as an internal standard. ^1^H NMR was carried out on a Jeol NMR spectrometer ECS-400 (Peabody, MA). Peaks were first corrected to a zero baseline compared to TMS, then compared against each spectrum (i.e., PCL and ACV before VASE treatment and PCL-ACV after VASE treatment).

### 2.5. Differential Scanning Calorimetry

Samples of untreated PCL, ACV, VASE-treated PCL, VASE-treated ACV, and VASE-created ACV:PCL powders were each heated from 25°C to 300°C on a Perkin Elmer DSC 8000 (Perkin Elmer, Waltham, MA) at 10°C/min. Three different samples for each material combination were tested.

### 2.6. Determining* In Vitro* Release Kinetics

Devices were submerged in 10 mL of 70% ethanol for 5 minutes twice in order to surface-sterilize them. Then devices were submerged in 10 mL of Dulbecco's phosphate-buffered saline without calcium or magnesium (DPBS; Corning CellGro, Tewksbury, MA) four times, the first three times for 10 minutes each and the fourth time for five minutes. ACV-containing SID- and VASE-created devices (7-mm × 2-mm) containing 30:70 drug (w:w) were placed two per well in a 24-well tissue culture plate with 1 mL of DPBS (“release medium”) per well at 37°C, 5% CO_2_ in a humidified environment. SID- and VASE-created PCL devices without the addition of ACV were used as controls. Two 7-mm × 2-mm devices were used due to the size restriction of a 24-well tissue culture plate, while maintaining the same surface area of one 15-mm × 2-mm device. Release medium was collected and replaced with fresh DPBS once every 24 hours for 60 days. ACV concentrations were determined by HPLC, as described below.

In a second set of experiments, two 7-mm × 2-mm ACV-containing VASE-created devices, of varying drug ratios, and SID-created devices (30:70 (w:w) ACV:PCL) were assayed in triplicate in a 12-well Transwell plate (Corning Costar, Kennebunk, ME) with HSV-1-infected Vero cells (1 × 10^5^ Vero cells [ATCC CCL-81] per well) in 2 mL complete DMEM (Dulbecco's modification of minimal essential medium [Corning CellGro] with 10% FBS [Hyclone/GE Healthcare Life Sciences, Logan, UT], 1% Glutamax [Gibco/Thermo-Fisher, Grand Island, NY], and 1% antibiotic/antimycotic [Corning CellGro]) in a 37°C incubator at 5% CO_2_. Briefly, cells were plated on day one. Devices were fabricated and sterilized as above, then placed in the Transwells on the second day of the experiment. Control treatments (medium with 25 *μ*g/mL ACV and medium with no ACV) were added to another set of Transwells; 25 *μ*g/mL ACV is well above any inhibitory concentration on HSV-1* in vitro* [30]. A one day pretreatment allowed for some ACV to enter cells and provided at least a modicum of prophylaxis. Cells were infected with 4 × 10^5^ pfu HSV-1 (KOS) (ATCC VR-1493) per well on day three of the experiment for a multiplicity of infection (MOI) of approximately 1 (see Antiviral Efficacy, below). After 30 hours the entire 1 mL of medium was collected and stored at -20°C. Non-infected cells were then assayed for viability (see Assessment of toxicity, below). An aliquot (100 *μ*L) of the saved medium was used to assay ACV levels by HPLC; another 200 *μ*L was used to determine HSV-1 titers by qPCR (see Antiviral Efficacy, below).

### 2.7. High Pressure Liquid Chromatography (HPLC)

Acetonitrile (900 *μ*L) was added to each 100 *μ*L aqueous sample (DPBS or DMEM) for drug release determination by HPLC as previously described [26, 27]. The amount of drug in each sample was determined through HPLC and UV spectrophotometry against a 15-point standard curve, as previously described [26, 27].

### 2.8. Assessment of Cytotoxicity

A representative field of each treatment with or without infection was photographed with an iPhone 6S Plus through a 10x ocular lens on an Olympus CK40 inverted microscope at a total magnification of 200x.Final images were imported into Adobe Photoshop CC 2017 for Mac OS X, where they were downsampled to greyscale, and contrast and brightness were normalized across all images. The final figure was assembled in Adobe Illustrator CC 2017 (Mac OS X). One additional set of wells was initially filled with 2 mL complete DMEM alone, to serve as a background control for an MTT assay, and was incubated exactly as were all other Transwell experiments.

After each well was photographed, cells were subjected to a standard MTT cell viability assay as described [31]. Briefly, the medium from each well was removed, washed once with 1 mL DPBS, then cells were allowed to incubate in 400 *μ*L of a freshly prepared 0.5 mg/mL solution of thiazolyl blue tetrazolium bromide (MTT) (Alfa Aesar, Ward Hill, MA) in DPBS at 37°C/5% CO_2_ for 30 minutes. DMSO (800 *μ*L) was added to each well and the plates were shaken for 30 min. An aliquot of the solution (200 *μ*L) was then transferred to a 96-well plate, where the OD_570_ and OD_620_ were read on a VersaMax Tunable Microplate Reader (Molecular Devices, Sunnyvale, CA). For each sample the OD_620_ was subtracted from the OD_570_. To eliminate background, this difference was subtracted from the value calculated for the blank well that did not contain any cells. All three trials were then averaged per treatment condition and normalized by dividing the difference from the positive control (cells only with no VASE-created rods or ACV) to obtain a percent viability.

### 2.9. Antiviral Efficacy

For Transwell samples that were infected, HSV-1 (KOS) was diluted to the appropriate concentration in DMEM without serum. Medium was removed from each cell layer and saved, then 100 *μ*L of virus (4 × 10^5^ pfu diluted in DMEM) was added dropwise to each well. Plates were placed back at 37°C and gently agitated every 10 minutes for one hour to allow virus adsorption. The inoculum was removed from each well to remove any residual unbound virus. The medium that was collected before infection was then reintroduced to its corresponding well and infection was allowed to proceed for 30 hours, at which point the medium was collected again as outlined above.

The medium from these HSV-1-infected samples (200 *μ*L) or from serially diluted HSV-1 (KOS) standards (200 *μ*L in DPBS, starting at 1 × 10^6^ pfu/mL and following a 10-fold dilution series to a titer of 1 × 10^0^ pfu/mL) were used to determine antiviral efficacy by qPCR. Virus DNA was isolated via the QIAamp DNA Blood Mini Kit using the DNA Purification from Blood or Body Fluids protocol (Qiagen, Chatsworth, CA); each sample had an additional 1 *μ*L of glycogen (Thermo-Fisher) added at the start to enhance isolation of all the DNA in the sample..

Each qPCR reaction was composed of 7 *μ*L of dH_2_O, 1.25 *μ*L of HSV-1 gD forward primer (0.3 *μ*M; ATCCGAACGCAGCCCCGCTG [32]), 1.25 *μ*L of HSV-1 gD reverse primer (0.3 *μ*M; TCTCCGTCCAGTCGTTTATCTTC [32]), 12.5 *μ*L of Sybr Green master mix (Thermo-Fisher), and 3 *μ*L of DNA isolated by the protocol described above. Each qPCR reaction was pipetted into a 96 well PCR plate (BioExpress, Kaysville, UT), and covered using Polyolefin Sealing Film (BioExpress). The plate was placed in a CFX Connect Real-Time PCR Detection System (Bio-Rad, Hercules, CA), and the CFX Manager (Bio-Rad) program was set to 95°C for 10 minutes followed by 40 cycles of 95°C for 15 seconds, 60°C for 30 seconds, and 72°C for 30 seconds. Titers of unknown samples were correlated to 7-point standard curve. All calculated pfu/mL values remained in log form.

### 2.10. Statistical Analysis

Statistical analysis was performed utilizing one-way analysis of variance (one-way ANOVA) and a Tukey post hoc test. All values are given as mean ± standard deviation. A p value < 0.05 was considered statistically significant.

## 3. Results

### 3.1. Physical and Chemical Characterization of SID- and VASE-Created Rods

**Figure 1:**
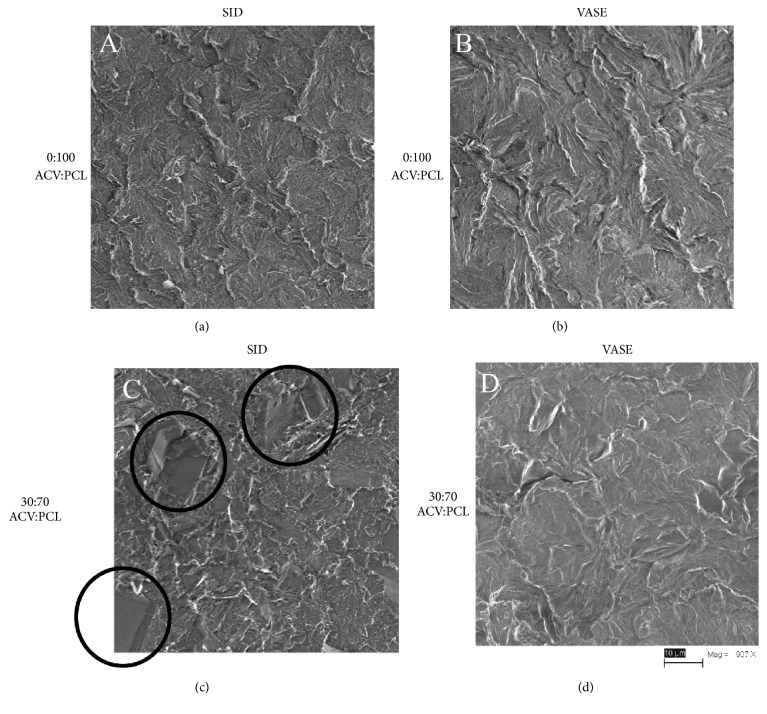
Scanning electron micrographs of SID-treated v. VASE-treated rods, cross sections. Scale bar at bottom right shows 907x magnification and 10 *μ* length.** (a) **PCL alone, treated by the SID method.** (b) **PCL alone, treated by the VASE method.** (c) **30:70 w:w ACV:PCL rod created by SID method; note black circles around large ACV crystals.** (d) **30:70 w:w ACV:PCL rod created by VASE method; note absence of large ACV crystals.

Cross sectioning and SEM were used to show differences between SID-created devices and VASE-created devices (Figure 1). PCL treated with the SID method (Figure 1(a)) and with the VASE method (Figure 1(b)) did not show any differences in the topological structure of the polymer. The wavy appearance of the polymer itself is also present in these micrographs. When the 30:70 (w:w) ACV:PCL SID-created device was imaged, large crystals were easily visible (Figure 1(c); black circles). Because the devices in Figure 1(c) differed from those in Figure 1(a) only by the presence of ACV, and because PCL exhibits a wavy pattern in Figures 1(a) and 1(b), the crystals in Figure 1(c) must be composed of ACV. The 30:70 (w:w) ACV:PCL VASE-created device (Figure 1(d)) lacks ACV crystals, indicating that a molecularly homogeneous distribution of drug has been achieved throughout the polymer matrix.

**Figure 2:**
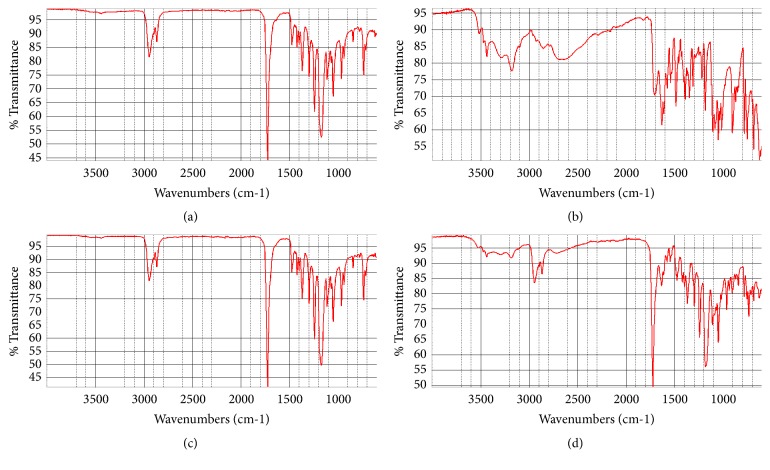
FTIR of materials in Figure 1.** (a)** untreated PCL;** (b)** untreated ACV;** (c)** SID-treated 0:100 (w:w) ACV:PCL;** (d)** SID-treated 30:70 (w:w) ACV:PCL.

SID-created devices were further characterized by FTIR. Samples of untreated PCL and ACV were analyzed to obtain spectra of the original materials. Samples of SID-created 0:100 (w:w) ACV:PCL and 30:70 (w:w) ACV:PCL, corresponding to the EM samples in Figure 1, were also subjected to FTIR for comparison (Figure 2).

Before SID treatment, pure PCL exhibits a classic ester IR stretch at 1777 cm^−1^ and standard alkane stretches at 2864 and 2942 cm^−1^ (Figure 2(a)); none of these are present in the FTIR of ACV (Figure 2(b)). Also before SID treatment, pure ACV shows stretches of 900 and 1628 cm^−1^, indicative of its conjugated ring system (Figure 2(b)); furthermore, ACV exhibits broad stretches at 2679 and 3436 cm^−1^, likely indicating the exchangeable protons.

FTIR showed that the SID process did not change the characteristics of PCL and showed that there were no leftover contaminants in devices composed of 0:100 (w:w) ACV:PCL from Figure 1(a) (Figure 2(c)). Every IR stretch that is visible in Figure 2(a) is seen in Figure 2(c), with no extraneous stretches present.  Figure 2(d) shows FTIR of SID-created 30:70 (w:w) ACV:PCL devices; while the same stretches in Figures 2(a) and 2(c) can be observed, the telltale stretches between 600 and 900 cm^−1^, 1500-1700 cm^−1^, 1850-2800 cm^−1^, and 3000-3600 cm^−1^ reveal the presence of ACV. There are no other chemical stretches visible in Figure 2(d), demonstrating that the only substances present in these devices are ACV and PCL.

To ensure that the treatments used in VASE did not compromise the structural integrity of the PCL, untreated PCL, SID-treated PCL, and VASE-treated PCL were ground into powder and subjected to gel permeation chromatography (Table 1). Both SID and VASE treatments only slightly reduced the Mn of PCL (less than 10% reduction). The Mw and polydispersity were also not appreciably changed (less than 8% reduction in Mw). These minor molecular weight shifts show that VASE treatment had little to no effect on the integrity of the average polymer chain length.

**Figure 3:**
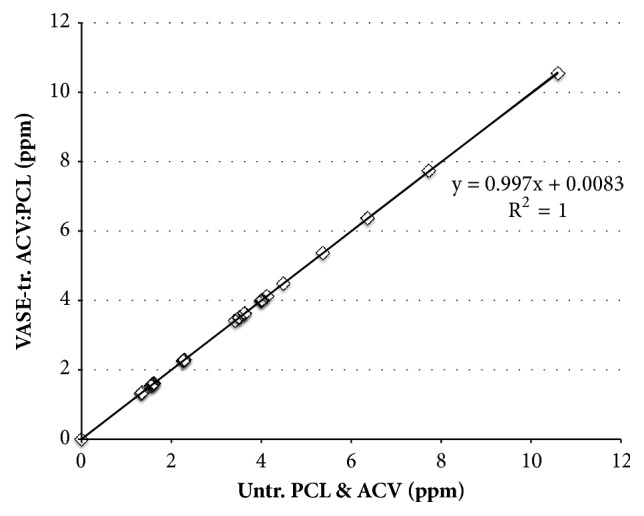
Comparison of ^1^H NMR of untreated (Untr.) ACV and PCL v. VASE-treated (VASE-tr.) ACV:PCL. Untreated ACV and PCL were subjected to ^1^H NMR and the chemical shifts recorded (x-axis; see Table 2). A sample of 30:70 w:w ACV:PCL that was VASE treated was also subjected to ^1^H NMR (y-axis); chemical shifts are also available in Table 2. For each ordered pair, the ppm of an identified peak in untreated material was matched to the ppm of a corresponding peak from treated material. The slope of the calculated best-fit line shows that the untreated and treated materials have almost identical peaks, and the R^2^ value shows that the line fits with almost perfect precision.

To ensure that VASE treatment did not compromise the chemical integrity of the device's components, powders of untreated ACV prepared in deuterated DMSO (Fig.  S1) and untreated PCL dissolved in deuterated acetonitrile (Fig.  S2) were each subjected to ^1^H NMR analysis and the chemical shift of each peak was recorded (Table 2, columns 1 and 2; Figure 3, x-axis). A powder of a combination of ACV and PCL was dissolved in 50:50 (v:v) mixture of deuterated DMSO:deuterated acetonitrile and also analyzed (Fig.  S3). Chemical shifts of peaks from a 30:70 (w:w) ACV:PCL device created through VASE, dissolved in a 50:50 (v:v) mixture of deuterated DMSO:deuterated acetonitrile, were also recorded (Fig.  S4, Table 2, column 3; Figure 3, y-axis). The chemical shift (in ppm) of each ^1^H peak from the untreated materials matched that of the VASE-treated materials (Table 1), as shown by the slope of the line (0.997) in the curve (Figure 3). The linear representation of the curve and the actual data points are well matched, indicating VASE treatment did not appreciably alter the ACV or PCL in the resulting devices.

Finally, PCL (VASE-treated or untreated), ACV (VASE-treated or untreated), and 10:90, 30:70, 50:50 (w:w) ACV:PCL VASE-created devices were ground into powders, then each was analyzed for its Tm and enthalpy of melting by differential scanning calorimetry (Table 3).

For PCL, heating revealed a significant difference in Tm between untreated PCL and 0:100 and 50:50 (w:w) VASE-treated ACV:PCL devices (Table 3; averages of 70.20°C v. 63.84°C (T = -4.50, p < 0.01); 70.20°C v. 65.01°C (p < 0.05), respectively), but not between untreated PCL and 10:90 and 30:70 (w:w) VASE-treated ACV:PCL devices (Table 3, averages of 70.20°C v. 65.60°C (T = -3.26, p > 0.05); 70.20°C v. 65.58°C (T = -3.27, p > 0.05), respectively). The Tm's among the VASE-treated PCL samples (Table 3) were similar (all p values > 0.05). As was seen with PCL, the Tm of untreated ACV (Table 3) was significantly higher than that of any ACV in VASE-treated materials (averages of 257.29°C v. 238.49°C (T = -6.61, p < 0.01); 257.29°C v. 233.87°C (T = -8.24, p < 0.01); 257.29°C v. 232.81°C (T = -8.61, p < 0.01); 257.29°C v. 233.82°C (T = -8.26, p < 0.01)). As with the VASE-treated PCL samples, the Tm differences among the VASE-treated ACVs were similar (all p values > 0.05).

Heating 100% PCL, whether VASE-treated or untreated, does not affect the enthalpy of melting (Table 3; 73.0775 J/g v. 75.3630 J/g). However, VASE-treated PCL doped with ACV has an enthalpy of melting that is inversely proportional to the ACV percentage. Similarly, as the ACV concentration increased in VASE-created devices, the drug's enthalpy of melting increased (Table 3).

### 3.2. Release Kinetics of SID- and VASE-Created Devices

**Figure 4:**
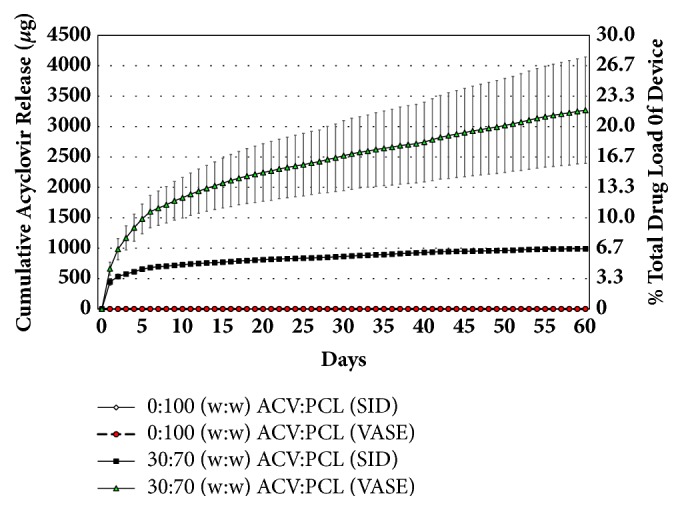
Release kinetics of ACV from SID-created v. VASE-created rods. Six 30:70 (w:w) ACV:PCL devices, created by the VASE method, and six 30:70 (w:w) ACV:PCL devices, created by the SID method were subjected to a 60-day trial examining release kinetics of ACV. Six VASE-created 0:100 (w:w) ACV:PCL devices and six SID-created 0:100 (w:w) ACV:PCL devices were used as a control. All devices were 7 mm × 2 mm; two were used per well to ensure identical surface area to one 15 mm × 2 mm device. Rods were placed in a 24-well plate and 1 mL DPBS was added. Each day for 60 days, that DPBS was removed and replaced. Each day's collected solution was subjected to HPLC to determine ACV concentrations, which were graphed additively to show a stepwise increase in total ACV release over time. Right y-axis shows cumulative drug release from devices as a percent of total drug load.

To determine the release kinetics of ACV, 30:70 (w:w) ACV:PCL SID-created devices and 30:70 (w:w) ACV:PCL VASE-created devices were subjected to a 60-day release kinetics trial in daily changes of 1 mL of DPBS (Figure 4). The 30:70 (w:w) ACV:PCL VASE-created devices showed a burst of drug release through day 6, then a relatively consistent and linear release of ACV over the rest of the 60-day period, with a cumulative release of ~3250 *μ*g of ACV over 60 days. The 30:70 (w:w) SID-treated devices exhibited one-third of the release of the VASE-treated devices, only releasing a total ~1000 *μ*g of ACV over the 60-day trial.

To compare the near zero-order nature of drug release over time, R^2^ values were obtained for a theoretical straight line of cumulative ACV release for devices made from each method. An R^2^ value of 0.8951 (F = 126.38) was obtained for ACV release from VASE-treated devices and 0.6991 (F = 27.47) for SID-treated devices for days 0 through 6 of the 60-day trial (data not shown), indicative of the less predictable burst release nature of the drug commonly seen in matrix-based delivery devices in both cases [33]. Additionally, VASE- and SID-treated devices displayed R^2^ values of 0.9752 (F = 14.00) and 0.9887 (F = 50.62) for drug release, respectively, after the first six days of this trial (data not shown). These latter regressions of almost perfect lines show that both VASE- and SID-created devices exhibit relatively linear levels of drug release per unit time after the first six days of burst release kinetics. Nonetheless, the VASE-crafted devices consistently released more drug per unit time (28.2 *μ*g/mL/day) than their SID-crafted counterparts (5.5 *μ*g/mL/day) and continued to release those levels of drug throughout the entire 60-day trial period, indicating a total longevity of nearly six months of consistent drug release. This prediction is based on the initial burst of drug release, the steady-state release over a 60-day period (Figure 4), and an approximation that the expected drug delivery is 60% of the total drug load, based on calculations on small molecule drug delivery according to Fick and Higuchi [34].

### 3.3. Safety and Efficacy of VASE-Created Devices* In Vitro*

**Figure 5:**
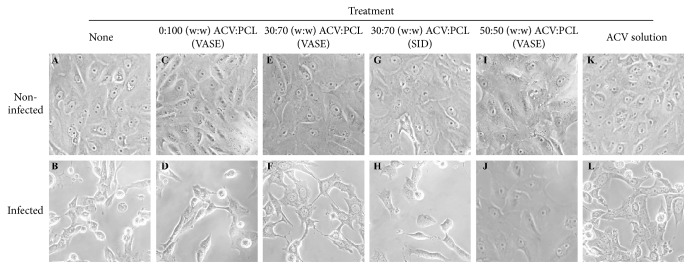
Photos of HSV-1infected Vero cells. Each photo is representative of a common field observed for each treatment that was conducted in triplicate. Treatment groups:** (A-B) **Nontreated controls;** (C-D) **devices containing 0:100 (w:w) ACV:PCL (VASE).** (E-F) **devices containing 30:70 (w:w) ACV:PCL (VASE). (**G-H)** devices containing 30:70 (w:w) ACV:PCL (SID). (**I-J)** devices containing 50:50 (w:w) ACV:PCL (VASE).** (K-L) **ACV solution at 25 *μ*g/mL. All photos in the top row show cells that were mock infected; photos in the bottom row show cells that were infected with HSV-1 (KOS) at an MOI of 1.

To determine the safety of VASE-created devices in cell culture, rods of ACV:PCL were created with varying concentrations of ACV. Devices were placed in the upper chambers of a 12-well Transwell plate with Vero cells in the lower chamber. Cells were infected with HSV-1 at an MOI of about 1. At 30 hours after infection, live cell images were taken using an iPhone 6S plus (Figure 5). Significant cytopathic effect (CPE) can be seen in samples that were left untreated (Figure 5(B)) and in control samples exposed to devices made with PCL alone (Figure 5(D)), whereas noninfected cells exhibit normal Vero cell morphology (Figures 5(A) and 5(C)). While VASE-crafted 30:70 (w:w) ACV:PCL devices (Figure 5(F)) seemed to provide slightly better protection than their SID-crafted counterparts (Figure 5(H)) or VASE-crafted devices containing no drug (Figure 5(D)), they were not as effective as VASE-crafted 50:50 (w:w) ACV:PCL devices (Figure 5(J)). Interestingly, these VASE-created 50:50 (w:w) ACV:PCL devices exhibited better protection than even 25 *μ*g/mL ACV directly added to the cell cultures (Figure 5(L)).

**Figure 6:**
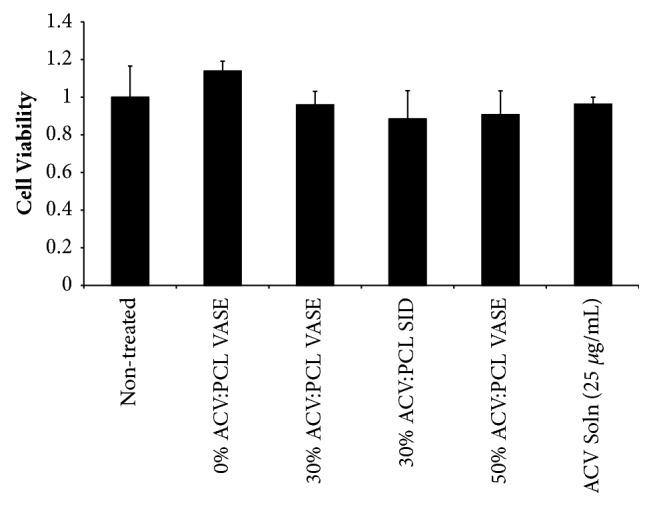
Assessment of cytotoxicity/safety of VASE-created devices. Experimental setup matched that of Figure 5. Thirty hours after infection, media was removed and cells were subjected to an MTT assay. Cell viability was determined by normalization against noninfected, nontreated controls. Data shown are mean ± standard deviation for experiment conducted in triplicate wells. Significance was confirmed using ANOVA and a Tukey post hoc test.

To ensure that all of these devices were not detrimental to cell viability, untreated cells and cell treated with different devices or ACV solution were subjected to an MTT cell viability assay (Figure 6). All treatment conditions were compared to a control of untreated Vero cells and no significant decrease in cell viability was observed; these data were confirmed via ANOVA (p = 0.159).

**Figure 7:**
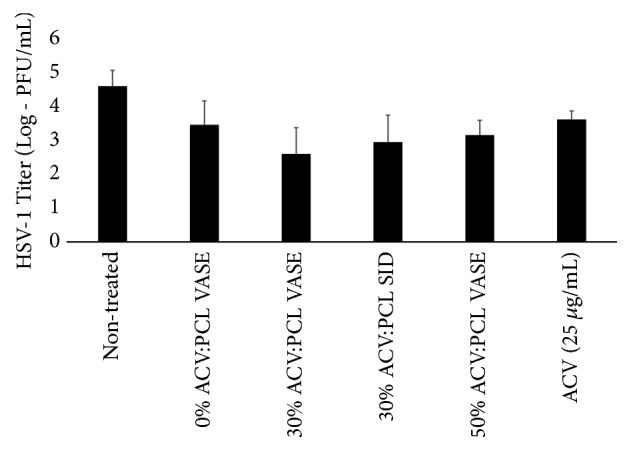
Suppression of HSV-1 infection of Vero cells* in vitro* by VASE- and SID-created devices. Experimental setup matched that of Figure 5. Thirty hours after infection, cell media was removed and HSV-1 viral DNA was isolated. Viral DNA was measured by qPCR. Data shown are means ± standard deviation for triplicate wells. Significance was confirmed using ANOVA and a Tukey post hoc test. Asterisks indicate values that were not significantly different from one another (p = 0.804). Asterisked values, though, are significantly different from the untreated control. (p < 0.05).

The antiviral efficacy of ACV:PCL devices was confirmed by quantifying the viral titer using qPCR for HSV-1 DNA from the cell culture media (Figure 7). Nontreated cells and cells treated with implants made of PCL alone exhibited the highest viral titer. All other ACV-containing, VASE-created rods (30:70 (w:w) ACV:PCL VASE, 30:70 (w:w) ACV:PCL SID, and 50:50 (w:w) ACV:PCL VASE) showed a significantly lower viral titer than was found in nontreated controls (Figure 7, asterisked columns; p < 0.001, T = 10.33; p = 0.001, T = 5.56; p < 0.001, T = 16.39, respectively). SID-treated 30:70 (w:w) ACV:PCL also showed a significantly lower viral titer than nontreated controls (Figure 7, p < 0.05), yet no significant difference was seen in comparing the protection provided by VASE and SID-created 30:70 (w:w) ACV:PCL devices (p = 0.336).

**Figure 8:**
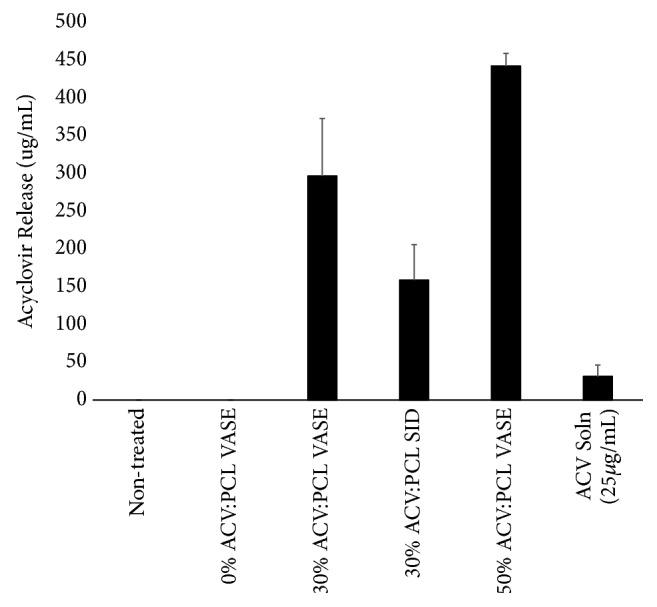
Drug release* in vitro* by VASE- and SID-created devices during infection of Vero cells with HSV-1. Experimental setup matched that of Figure 5. Thirty hours after infection, cell media was removed and ACV concentration was quantified by HPLC using an Agilent 1200 HPLC. Data shown are means ± standard deviation for triplicate wells.

ACV levels from the infection experiments were also quantified to determine both (1) the release of ACV from VASE- and SID-created devices using culture medium as the “release medium,” and (2) whether drug levels in the medium correlated with the level of virus suppression. Levels of ACV were quantified by HPLC, as above, and graphed as overall ACV release during experimentation (described in Figure 5). Devices containing a higher drug:polymer ratio released comparatively more ACV (Figure 8), and these increased levels of ACV in culture correlated with HSV-1 suppression (Figures 5 and 7). Nonetheless, increased drug load (Figure 8) did not always correlate with increased viral suppression (Figure 7); for example, devices composed of VASE-created 30:70 (w:w) ACV:PCL released comparatively less drug than those containing 50:50 (w:w) ACV:PCL (Figure 8), yet no statistically different decrease in viral titer was observed (Figure 7).

## 4. Discussion

Although individuals with HSV-1 are typically asymptomatic, recurrent infections and infections in immunocompromised patients require active therapeutic intervention due to the difficulty of controlling the virus [1, 7–9]. While oral dosing of nucleoside analogues (e.g., ACV and PCV) is the most common therapeutic approach, poor oral bioavailability [10] and short* in vivo *half-life [10] in the face of poor patient compliance [12, 15] lead to subsequent decreased antiviral efficacy [12, 20]. Poor suppression can eventually lead to more drug-resistant mutants [19, 20]. Our work shows a more effective long-term delivery system in which the antiherpetic drug ACV was combined with the bioerodable polymer polycaprolactone in a homogenous drug:polymer mixture.

SEM showed that there is a visual difference between the nonhomogeneous SID-created devices and the molecularly homogeneous VASE-created devices (Figure 1). Devices fabricated by the SID method are not molecularly homogeneous because the ACV probably remained in a colloidal suspension during solvent evaporation. This resulted in pockets of drug in the polymer matrix that led to less uniform drug release kinetics and an uneven bulk erosion of the device, likely caused by nonuniform solvation of large ACV crystals (Figure 1(c)). Materials created via the VASE method are molecularly homogeneous due to acid solubilization of the acyclovir in an acetonitrile solution, creating an even distribution of drug and polymer. This even distribution probably provided consistent surface erosion, as opposed to bulk erosion, throughout the lifetime of the devices [33].

VASE treatment did not appear to alter the characteristics of ACV or the PCL matrix appreciably (Tables 1-3, Figures 1–3). It is possible that the apparent decrease in Tms (Table 3) may have occurred because both drug and polymer are still protonated from the formic acid treatment; while the protonation would most likely not significantly affect drug or polymer performance, it may simply lower the Tm of each material. Similarly, while the enthalpy of melting appears to lower when ACV content is increased (Table 3), this decrease is most likely caused by fewer PCL molecules being adjacent to one another in the final materials, decreasing any crystal structure they may have between identical molecules [34]. It was also probable that more ACV molecules were adjacent to each other and able to form more ordered ACV structures. Interestingly, and unlike our observations with VASE treatment of PCL, pure ACV treated in this way has a lower enthalpy of melting than untreated, pure ACV.

The consistent daily release of ACV from 30:70 (w:w) VASE-created devices achieved levels that ranged from 663.28 *μ*g/mL ± 106.01 (on day one) to 19.08 ± 9.96 *μ*g/mL (on day 60) (Figure 4). These levels never drop below 14.93 *μ*g/mL after the initial release of ACV, and are well above the 0.02-0.9 *μ*g/mL concentrations required to inhibit HSV-1 in cell culture systems [10].* In vivo* ACV concentrations in plasma reach 0.7-9.8 *μ*g/mL after 5 mg/kg intravenous doses every 8 hours, and levels average concentrations of 5-6 *μ*g/mL after a single 1000 mg oral dose of valacyclovir [10]. Therefore, although released in a limited volume* in vitro*, VASE-created devices provide a comparable and consistent ACV level well above what is required for suppression.

VASE-created devices produced more consistent daily drug release versus the plateaued drug release from those created through the SID method (Figure 4). Furthermore, VASE-created devices showed a higher total release of ~3.2 mg of ACV (v. ~1 mg of ACV for SID-fabricated materials of comparable drug load), and VASE-treated 30:70 (w:w) ACV:PCL devices would likely have lasted for at least 15 months given the drug load in that series of materials. SID-treated devices also exhibited a much more erratic release rate within the first 6 days of drug delivery, and ACV release from those devices appeared to nearly halt by day 60. Therefore, in addition to the VASE method not having any effect on PCL integrity, this fabrication method appears to lead to a more stable, consistent, longer-term release of ACV.

Importantly, we show that VASE-created devices of adequate ACV loads suppressed primary HSV-1 infection (Figures 5 and 7) while still being nontoxic to the host cells (Figures 5 and 6). While PCL and its breakdown products normally exhibit low toxicity [22, 23] and ACV also has a high therapeutic index [11, 18], we could not reasonably predict that the acetonitrile and formic acid levels of VASE-created materials would be low enough to prevent host cell death. We were able to detect such trace amounts of formic acid, as evidenced by the 8.1 ppm peak in the ^1^H NMR analysis (Fig.  S4). This trace amount represented less than 0.2% of the total mass of protons in the sample, and therefore cytotoxicity remained a possibility. However, the VASE methodology clearly reduced the formic acid amount to a nontoxic level, as Vero cells easily thrived in culture in the presence of VASE-treated devices (Figures 5 and 6).

SID-treated 30:70 (w:w) ACV:PCL devices released significantly less ACV than VASE-treated 30:70 (w:w) ACV:PCL devices (Figures 4, and 8, p = 0.005, T = -4.77), yet still no difference in viral titer was observed (Figure 7). There is probably a level of drug:polymer ratio at which drug-drug and drug-polymer interactions significantly alter release kinetics, to the point that the drug load is no longer directly correlated with effective delivery; this phenomenon has been observed in other matrix-based controlled release devices [35]. Therefore, a range of drug:polymer ratios may be useful for HSV-1 protection.

The development of a controlled release subcutaneous implant would prevent the main issues of orally delivered ACV, including the low oral bioavailability [10], short half-life [10], and need for patient compliance [12, 15]. Negating the issue of patient compliance is paramount [12, 15], since any variance in administration could result in drug troughs. If drug levels drop below an inhibitory threshold the virus might replicate, which could result in the spontaneous appearance of drug-resistant mutants [10]. By releasing a consistent ACV concentration of about 14 *μ*g/mL, a dose well above the average concentrations of a 5 *μ*g/mL oral dose of valacyclovir may be achievable. This drug delivery system would administer constant doses of ACV so drug levels would stay above the therapeutic threshold in tissues. Because of its presumably local targeting that avoids the need for gut absorption, ACV administered by these devices would lead to a lower daily dose and increased bioavailability. With the homogeneity of the novel VASE-created devices, we expect surface erosion instead of bulk erosion, causing improved stability, longevity of the vehicle, and improved kinetics of drug release [36].

While the ACV:PCL subcutaneous implants only explore one avenue of drug release, there has been concerted effort to create ACV delivery systems through other routes of administration (topical, oral, intravenous) and other systems that do not involve a polymer:drug matrix for successful drug delivery. The use of vesicular drug delivery systems (niosomes and liposomes) have been very successful with both topical and oral delivery of ACV [37–40]. Yet these drug delivery schemes deliver ACV too rapidly; 90% of acyclovir in liposomes and 50% of acyclovir in niosomes is released in 150 and 200 minutes, respectively [41], and therefore cannot be utilized as long-term delivery systems. Micro- and nanoparticles composed of PLGA microspheres and PLA nanospheres and integration incorporating ACV via solvent evaporation have also been explored [42, 43]. Nonetheless, as observed with the vesicular drug systems, microparticle drug delivery systems have a relatively fast drug delivery time of 10-12 h, and have yet to be examined as long-term ACV drug delivery systems [41]. In comparison with VASE-created ACV:PCL devices, vesicular and microparticle delivery systems incorporate less ACV (only as high as 10% of the delivery system), but have substantially reduced longevity when compared to the system described here. Therefore, while other drug delivery systems can be used to administer ACV over the course of minutes and hours, VASE-created ACV:PCL devices have the potential to deliver ACV for nearly six months.

Future studies will test the safety and efficacy of VASE-created devices with a higher molecular weight PCL, which may change longevity and/or drug release kinetics [44, 45]. We will also test the breakdown of PCL over time, to observe if SID- and VASE-created devices have altered PCL frameworks, and if this accounts for the difference in drug release and the observed crystallization of acyclovir. Drug release and longevity might also be improved with a differently shaped device. While rods are a common shape for controlled release devices, disks, ellipsoids, and other common device geometries should be investigated. It is also important to investigate whether other treatments for HSV-1, such as penciclovir, can be incorporated into controlled release vehicles created using the VASE method. If the VASE method can be applied to other acid-stable drugs, this matrix construction methodology may revolutionize drug delivery systems for countless other systems.

In the current study, we established efficacy of the device against infection with HSV-1 in culture. The efficacy of these types of devices in treating other herpesviruses, especially HSV-2 and varicella zoster virus (VZV), which are common herpesviruses typically responsible for genital herpes and shingles, respectively [46], still needs to be determined. By determining whether different drugs can be used in these devices and whether the devices can be used to treat different types of herpesviruses, we can establish just how far-reaching this methodology may be. We expect that the homogeneity provided by the VASE method will result in materials with longer life, better release characteristics, and better integrity of any implantable materials over time.

## 5. Conclusions

This study demonstrated that ACV can successfully be incorporated into a PCL-based, bioerodable controlled release device that is capable of delivering functional ACV at steady rates over an extended time period. The methods used to fabricate these devices (VASE) resulted in molecularly homogeneous distribution of ACV throughout the matrix without disturbing chemical integrity of the drug or matrix material. VASE also leads to better near zero-order drug release characteristics and likely surface, instead of bulk, erosion of the polymer matrix throughout the lifetime of the device. Finally, these materials safely and successfully prevent primary infection of HSV-1 in Vero cells, demonstrating their eventual utility as clinically deployable antiherpetic measures.

## Figures and Tables

**Table 1:** GPC comparison of untreated PCL v. treated PCL.

	untr. PCL	SID-tr. PCL	VASE-tr. PCL
M_n_	18294	17264	17105
M_w_	25656	25147	23772
PDI	1.402412	1.45663	1.389758

untr. PCL: untreated PCL.

SID-tr. PCL: PCL treated by SID method.

VASE-tr. PCL: PCL treated by VASE method.

Mn: number average molecular weight.

Mw: weight average molecular weight.

PDI: polydispersity.

**Table 2:** ^1^H NMR comparison of chemical shifts of untreated ACV and PCL v. treated ACV:PCL.

	Chemical Shift in NMR (ppm)		
	untr. PCL	untr. ACV	VASE-tr. ACV:PCL
TMS	0	0	0

PCL	1.3230		1.3230
	1.3471		1.3471
	1.5418		1.5418
	1.5613		1.5613
	1.5796		1.5796
	1.5957		1.5957
	1.6129		1.6129
	2.2566		2.2555
	2.2749		2.2749
	2.2933		2.2933
	3.4124		3.4124
	3.6163		3.6163
	3.6278		3.6289
	3.9840		3.9840
	4.0001		4.0001
	4.0172		4.0172
	4.1192		4.1192

ACV		3.5006	3.4983
		3.5075	3.5052
		4.4846	4.4852
		5.3609	5.3586
		6.3529	6.3609
		7.7252	7.7263
		10.5924	10.5351

untr. PCL: untreated PCL.

untr. AC:, untreated ACV.

VASE-tr. ACV:PCL: 30:70 (w:w) ACV:PCL treated by VASE method.

TMS: tetramethylsilane internal standard.

**Table 3:** Tm and ΔH comparison of untreated ACV and PCL v. treated ACV-PCL by DSC.

Material	Tm (°C)	ΔH (J/g)
untr. PCL	70.20 ± 0.10	73.0775 ± 1.3384
VASE-tr. 0:100 (w:w) ACV:PCL	63.84 ± 0.43	75.3630 ± 4.7970
VASE-tr. PCL in 10:90 (w:w) ACV:PCL device	65.60 ± .030	69.9022 ± 1.5858
VASE-tr. PCL in 30:70 (w:w) ACV:PCL device	65.58 ± 1.56	53.2311 ± 8.3285
VASE-tr. PCL in 50:50 (w:w) ACV:PCL device	65.01 ± 0.70	19.6963 ± 7.8685

VASE-tr. ACV in 10:90 (w:w) ACV:PCL device	238.49 ± 7.72	14.9521 ± 7.6689
VASE-tr. ACV in 30:70 (w:w) ACV:PCL device	233.87 ± 0.91	26.7761 ± 19.9455
VASE-tr. ACV in 50:50 (w:w) ACV:PCL device	232.81 ± 0.30	53.5217 ± 9.5136
VASE-tr. 100:0 ACV:PCL	233.82 ± 0.20	55.5956 ± 3.7339
untr. ACV	257.29 ± 0.34	143.5112 ± 23.3110

untr.: materials left untreated.

VASE-tr.: materials treated by VASE method.

Tm: melting point.

ΔH: enthalpy of melting.

## Data Availability

All raw data generated in this research are available by request from the authors. The data presented in this manuscript show the typical appearances (for photographs) or averages and standard deviations (for quantitative measures) of all the raw data collected.

## Supplementary Materials

Supplementary 1 Figure S1. ^1^H NMR of untreated ACV.

Supplementary 2 Figure S2. ^1^H NMR of untreated PCL.

Supplem entary 3 Figure S3. ^1^H NMR of untreated ACV:PCL mixture.

Supplementary 4 Figure S4. ^1^H NMR of VASE-treated 30:70 (w:w) ACV:PCL.
